# ﻿A new species of *Cyrtodactylus* Gray, 1827 (Squamata, Gekkonidae) from Yunnan Nangunhe National Nature Reserve, China

**DOI:** 10.3897/zookeys.1223.137184

**Published:** 2025-01-06

**Authors:** Shuo Liu, Zhimin Li, Wenguang Duan, Mian Hou, Dingqi Rao

**Affiliations:** 1 Kunming Natural History Museum of Zoology, Kunming Institute of Zoology, Chinese Academy of Sciences, Kunming, Yunnan 650223, China; 2 Yunnan Nangunhe National Nature Reserve Management and Protection Bureau, Cangyuan, Yunnan 677400, China; 3 College of Continuing (Online) Education, Sichuan Normal University, Chengdu, Sichuan 610066, China; 4 Kunming Institute of Zoology, Chinese Academy of Sciences, Kunming, Yunnan 650201, China

**Keywords:** Bent-toed gecko, Cangyuan, *Cyrtodactyluschauquangensis* group, mitochondrial DNA, systematics, taxonomy

## Abstract

A new forest-dwelling species of the *Cyrtodactyluschauquangensis* group is described from southwestern Yunnan Province, China. Phylogenetically, it was recovered as the sister species of *C.zhenkangensis*, with a genetic distance of 9.2% in the ND2 gene. Morphologically, the new species can be separated from *C.zhenkangensis* by the discontinuity of enlarged femoral scales and enlarged precloacal scales, the absence of femoral pores, and the difference in dorsal color pattern. In addition, although the new species and *C.zhenkangensis* are distributed relatively close, their habitats are clearly different. *Cyrtodactyluszhenkangensis* inhabits karst limestone, while the new species inhabits forest. The new species is the 29^th^ species of the *C.chauquangensis* group and the third forest-dwelling species of this group.

## ﻿Introduction

The species diversity of the genus *Cyrtodactylus* Gray, 1827 had previously been severely underestimated, but currently the number of species in this genus has been rapidly increasing, and the number of species is now over 350 (Uetz et al. 2024). In China, only two species of this genus were recorded before 2010 (Zhao et al. 1999, 2000), namely *C.khasiensis* (Jerdon, 1870) from Yunnan Province and *C.tibetanus* (Boulenger, 1905) from Xizang Autonomous Region. Subsequently, *C.zhaoermii* Shi & Zhao, 2010 was described from Xizang, and *C.wayakonei* Nguyen, Kingsada, Rösler, Auer & Ziegler, 2010 was reported from Yunnan (Yuan and Rao 2011). Later, *C.khasiensis* was removed from the herpetofauna of China, and *C.khasiensiscayuensis* Li, 2007 was elevated to the level of species (Agarwal et al. 2018; Wang et al. 2020). Soon after, *C.dianxiensis* Liu & Rao, 2021, *C.zhenkangensis* Liu & Rao, 2021, *C.gulinqingensis* Liu, Li, Hou, Orlov & Ananjeva, 2021, *C.hekouensis* Zhang, Liu, Bernstein, Wang & Yuan, 2021, and *C.menglianensis* Liu & Rao, 2022 were described from Yunnan, and *C.arunachalensis* Mirza, Bhosale, Ansari, Phansalkar, Sawant, Gowete & Patel, 2021 and *C.kamengensis* Mirza, Bhosale, Thackeray, Phansalkar, Sawant, Gowande & Patel, 2022 were described from near the border between China and India. Recently, *C.caixitaoi* Liu, Rao, Hou, Wang & Ananjeva, 2023 was described from Yunnan, *C.wayakonei* was removed from the herpetofauna of China (Liu et al. 2023), *C.hekouensis* was treated as a junior synonym of *C.gulinqingensis* (Zhang et al. 2024), and *C.laevis* Ma, Wang & Jiang, 2024 was described from Xizang. So far, 11 species of this genus have been recorded in China, including six species from Xizang and five species from Yunnan (Ma et al. 2024).

Yunnan Nangunhe National Nature Reserve is located in southwestern Yunnan Province, China. This nature reserve has a total area of 508.87 km^2^, with the lowest and highest elevations at 510 m and 2,977 m, respectively. There are numerous high mountains and valleys and many rivers and streams, as well as multiple vegetation types, such as rainforest and seasonal rainforest, in this nature reserve. Its main protected species are rare and endangered wild animals such as elephants, tigers, gibbons, and monkeys (Yang and Du 2004; Tang et al. 2015).

During our fieldwork in southwestern Yunnan, China, in 2024, two specimens of *Cyrtodactylus* were collected in Yunnan Nangunhe National Nature Reserve. Both morphological and phylogenetic analyses support the recognition of the two specimens as belonging to an unnamed species of the *C.chauquangensis* group. Therefore, we describe them as a new species below.

## ﻿Materials and methods

### ﻿Sampling

The field survey in Yunnan Nangunhe National Nature Reserve was conducted under the permit from Yunnan Nangunhe National Nature Reserve Management and Protection Bureau. Specimens were collected by hand at night and photographed alive prior to preservation. Liver tissues were dissected and preserved in analytical pure ethanol. The specimens were stored in 75% ethanol and deposited at Kunming Natural History Museum of Zoology, Kunming Institute of Zoology, Chinese Academy of Sciences (**KIZ**).

### ﻿Morphological analyses

Measurements were taken with digital calipers to the nearest 0.1 mm. Bilateral scale counts are given as left/right. The methodology of measurements and meristic counts is the same as those in Liu et al. (2023):

**AG** axilla to groin distance, measured from the posterior margin of the forelimb insertion to the anterior margin of the hindlimb insertion;

**DTR** dorsal tubercle rows, the number of dorsal, longitudinal rows of the tubercles at midbody between ventrolateral folds;

**ED** ear diameter, the greatest diameter of ear opening;

**EE** eye to ear distance, measured from the posterior edge of the orbit to the anterior edge of the ear opening;

**EFS** enlarged femoral scales, the number of the enlarged femoral scales beneath each thigh;

**ForeaL** forearm length, measured from the tip of the elbow to the wrist;

**FP** femoral pores;

**GSDT** granular scales surrounding dorsal midbody tubercles;

**HH** maximum head height;

**HL** head length, measured from the tip of the snout to the posterior margin of the retroarticular process of the lower jaw;

**HW** maximum head width;

**I** internasals, the number of the scales between the two supranasals;

**IFL** infralabials, counted from the first labial scale to the corner of the mouth;

**IND** internarial distance, the distance between nares;

**IOD** interorbital distance, measured across the narrowest point of the frontal bone;

**LF4** subdigital lamellae under the fourth finger, counted from the base of the digit where it contacts the body of the hand to the base of the claw, including the claw sheath;

**LT4** subdigital lamellae under the fourth toe, counted from the base of the digit where it contacts the body of the foot to the base of the claw, including the claw sheath;

**ML** mental length, the maximum length of the mental;

**MW** mental width, the maximum width of the mental;

**OD** greatest diameter of orbit;

**PAT** postcloacal tubercles, the number of the tubercles on each side of the postcloacal region;

**PM** postmentals, the number of the scales bordering the mental shield, excluding infralabials;

**PP** precloacal pores;

**PVT** paravertebral tubercles, counted in a single paravertebral row from the level of the forelimb insertions to the level of the hind limb insertion;

**RH** rostral height, the maximum height of the rostral;

**RW** rostral width, the maximum width of the rostral;

**SE** snout to eye distance, measured from the tip of the snout to the anterior edge of the orbit;

**SL** shank length, measured from the base of the heel to the knee;

**SPL** supralabials, counted from the first labial scale to the corner of the mouth;

**SVL** snout–vent length, measured from the tip of the snout to the anterior margin of the cloaca;

**TaL** tail length, measured from the posterior margin of the cloaca to the tip of the tail;

**V** ventral scale rows, the number of longitudinal rows of ventral scales at midbody between ventrolateral folds.

Morphological comparisons were based on the original descriptions of each species of the *Cyrtodactyluschauquangensis* group (Hoang et al. 2007; Bauer et al. 2009, 2010; Ngo and Grismer 2010; Nguyen et al. 2010, 2014, 2015, 2017; Sumontha et al. 2010; Luu et al. 2011; Ngo 2011; Ngo and Chan 2011; Kunya et al. 2014; Nazarov et al. 2014; Pauwels et al. 2014; Schneider et al. 2014, 2020; Le et al. 2016; Pham et al. 2019; Liu and Rao 2021, 2022; Liu et al. 2021, 2023; Chomdej et al. 2022; Grismer et al. 2024; Tran et al. 2024).

### ﻿Molecular analyses

Total genomic DNA was extracted from liver tissue samples. A fragment of the mitochondrial NADH dehydrogenase subunit 2 (ND2) gene was amplified and sequenced using the primers L4437b (5′-AAGCAGTTGGGCCCATACC-3′) and H5540 (5′- TTTAGGGCTTTGAAGGC -3′) (Macey et al. 2000) for the two newly collected specimens. Sequences were assembled and edited using SeqMan in Lasergene 7.1 (Burland 2000) and MEGA 11 (Tamura et al. 2021). New sequences have been deposited on GenBank and available sequences of the *C.chauquangensis* group were obtained from GenBank (Table 1). *Cyrtodactylusdammathetensis* Grismer, Wood, Thura, Zin, Quah, Murdoch, Grismer, Lin, Kyaw & Lwin, 2017 and *C.sinyineensis* Grismer, Wood, Thura, Zin, Quah, Murdoch, Grismer, Lin, Kyaw & Lwin, 2017 were used as outgroups following Grismer et al. (2024).

**Table 1.** Sequences (ND2) used in the phylogenetic analysis of this study.

Species	Locality	Catalog number	Accession number
*Cyrtodactylusnangunhe* sp. nov.	China, Yunnan, Lincang, Cangyuan	KIZ 2024083	PQ670135
*Cyrtodactylusnangunhe* sp. nov.	China, Yunnan, Lincang, Cangyuan	KIZ 2024084	PQ670136
*Cyrtodactylusauribalteatus*	Thailand, Phitsanulok, Noen Maprang	AUP-01745	MZ439914
*Cyrtodactylusbichnganae*	Vietnam, Son La, Son La Urban	UNS 0473	MF169953
*Cyrtodactylusbobrovi*	Vietnam, Hoa Binh, Lac Son	IEBR A.2015.29	MT953471
*Cyrtodactyluschauquangensis*	Vietnam, Nghe An, Quy Hop	NA 2016.1	MT953475
*Cyrtodactyluscucphuongensis*	Vietnam, Ninh Binh, Cuc Phuong NP	CP 17.02	MT953477
*Cyrtodactylusdoisuthep*	Thailand, Chiang Mai, Doi Suthep	AUP-00777	MT497801
*Cyrtodactylusdumnuii*	Thailand, Chiang Mai, Chiang Dao	AUP-00769	MT497802
*Cyrtodactylusdumnuii*	Thailand, Chiang Mai, Chiang Dao	AUP-00770	MT497803
*Cyrtodactylusdumnuii*	Thailand, Chiang Mai, Chiang Dao	AUP 00768	MW713972
*Cyrtodactyluserythrops*	Thailand, Mae Hong Son, Pang Mapha	AUP-00771	MT497806
*Cyrtodactylusgulinqingensis*	China, Yunnan, Maguan, Gulinqing	KIZ 061813	MZ782150
*Cyrtodactylushouaphanensis*	Laos, Luang Houaphan	IEBR A.2013.109	MW792067
*Cyrtodactylushuongsonensis*	Vietnam, Ha Noi, My Duc, Huong Son	IEBR A.2011.3A	MT953481
*Cyrtodactyluskunyai*	Thailand, Loei, Nong Hin	AUP-01747	MZ439916
*Cyrtodactylusluci*	Vietnam, Lao Cai, Bac Ha	IEBR R.5240	PP253960
*Cyrtodactylusmenglianensis*	China, Yunnan, Puer, Menglian	KIZ 20210713	OM296042
*Cyrtodactylusmenglianensis*	China, Yunnan, Puer, Menglian	KIZ 20210714	OM296043
*Cyrtodactylusmenglianensis*	China, Yunnan, Puer, Menglian	KIZ 20210716	OM296044
*Cyrtodactylusngoiensis*	Laos, Luang Prabang, Ngoi	IEBR A.2013.110	MW792066
*Cyrtodactylusotai*	Vietnam, Son La, Van Ho, Na Bai	TBU 2017.2	MT953486
*Cyrtodactylusphamiensis*	Thailand, Chiang Rai, Mae Sai, Pha Mi	ZMKU R 01074	PP430583
*Cyrtodactylusphukhaensis*	Thailand, Nan, Pua, Doi Phu Kha	AUP-01823	MZ439912
*Cyrtodactylusphukhaensis*	Thailand, Nan, Pua, Doi Phu Kha	AUP-01824	MZ439913
*Cyrtodactyluspuhuensis*	Vietnam, Thanh Hoa	ND 01.15	MT953489
*Cyrtodactylussolaensis*	Vietnam, Son La, Phu Yen	IEBR A.2017.1	MT953492
*Cyrtodactylussoni*	Vietnam, Ninh Binh, Gia Vien	IEBR R.2016.4	MT953491
*Cyrtodactylusspelaeus*	Laos, Vientiane, Kasi	HLM 0315	MW713962
*Cyrtodactylustaybacensis*	Vietnam, Son La, Quyun Nhai, Ca Nang	IEBR 4379	MT953495
*Cyrtodactylusvilaphongi*	Laos, Luang Prabang, Luang Prabang	IEBR A.2013.103	MT953497
*Cyrtodactyluswayakonei*	Laos, Luang Nam Tha, Vieng Phoukha	ZFMK 91016	MT953498
*Cyrtodactyluszhenkangensis*	China, Yunnan, Lincang, Zhenkang	KIZ L2020047	MW792062
*Cyrtodactyluszhenkangensis*	China, Yunnan, Lincang, Zhenkang	KIZ L2020048	PQ670137
*Cyrtodactylusdammathetensis*	Myanmar, Mon State, Mawlamyine	LSUHC:12863	MF872277
*Cyrtodactylussinyineensis*	Myanmar, Kayin State, Hpa-an	LSUHC:12836	MF872355

Sequences were aligned using MAFFT 7.471 (Katoh and Standley 2013) with default parameters. The best-fit substitution models (GTR+F+I+G4 for the first and second codon positions and the tRNAs, and GTR+F+G4 for the third codon position) were chosen using the Akaike information criterion (AIC) in ModelFinder (Kalyaanamoorthy et al. 2017). The technical computation methods for genetic divergences calculation and Bayesian-inference and maximum-likelihood phylogenetic analyses were the same as those used by Liu and Rao (2022).

## ﻿Results

**Figure 1.**
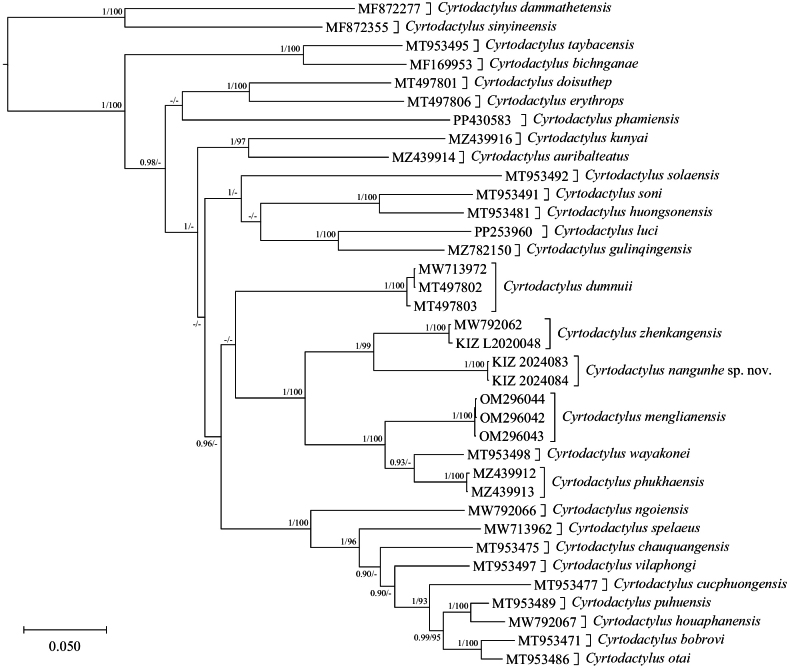
Bayesian phylogram of the *Cyrtodactyluschauquangensis* group based on the ND2 sequences. Numbers before slashes indicate Bayesian posterior probabilities and numbers after slashes indicate maximum-likelihood ultrafast bootstrap. The symbol “–” represents the value below 0.90/90.

The phylogenetic topologies of Bayesian-inference and maximum-likelihood analysis were identical. The sequences of the newly collected specimens were nested within the *Cyrtodactyluschauquangensis* group and formed a strongly supported lineage sister to *C.zhenkangensis* (Fig. 1). The genetic distance between the sequences of the newly collected specimens and the sequences of *C.zhenkangensis* was 9.2%, and the genetic distances between the sequences of the newly collected specimens and the sequences of other named species of this group ranged from 11.4% to 18.3% (Table 2).

**Table 2.** Uncorrected pairwise genetic distances (%) among species of the *Cyrtodactyluschauquangensis* group based on the ND2 sequences.

	1	2	3	4	5	6	7	8	9	10	11	12	13	14	15	16	17	18	19	20	21	22	23	24	25	26
1 *Cyrtodactylusnangunhe* sp. nov.																										
2 *Cyrtodactylusauribalteatus*	14.6																									
3 *Cyrtodactylusbichnganae*	18.3	18.0																								
4 *Cyrtodactylusbobrovi*	16.3	15.0	19.7																							
5 *Cyrtodactyluschauquangensis*	15.7	14.4	18.1	8.6																						
6 *Cyrtodactyluscucphuongensis*	16.8	15.7	19.9	7.9	8.5																					
7 *Cyrtodactylusdoisuthep*	16.8	14.1	16.4	15.4	14.2	15.2																				
8 *Cyrtodactylusdumnuii*	13.6	13.3	16.9	13.7	12.2	14.2	14.0																			
9 *Cyrtodactyluserythrops*	15.9	14.7	16.7	14.8	13.7	15.1	10.9	13.7																		
10 *Cyrtodactylusgulinqingensis*	15.7	14.5	18.1	13.7	14.0	14.0	13.6	12.9	14.1																	
11 *Cyrtodactylushouaphanensis*	16.3	15.6	19.4	6.5	9.0	7.5	15.1	14.2	15.1	14.0																
12 *Cyrtodactylushuongsonensis*	15.3	14.5	17.7	14.3	12.2	14.3	14.6	14.0	14.4	12.4	14.8															
13 *Cyrtodactyluskunyai*	16.5	12.5	17.8	15.5	14.4	17.1	14.9	13.8	15.7	14.4	16.3	14.7														
14 *Cyrtodactylusluci*	16.5	13.9	18.1	14.2	14.2	14.6	13.9	13.3	15.1	9.1	14.4	12.4	14.2													
15 *Cyrtodactylusmenglianensis*	11.5	12.6	18.4	14.9	12.7	15.0	15.1	11.4	14.4	14.3	14.7	14.4	15.0	14.0												
16 *Cyrtodactylusngoiensis*	15.4	13.8	18.2	11.2	10.3	10.8	14.8	12.0	14.5	13.1	11.4	13.1	13.8	13.4	13.1											
17 *Cyrtodactylusotai*	15.9	14.1	19.1	3.6	9.1	8.4	16.2	15.6	16.4	15.6	6.8	14.7	15.5	14.8	15.2	12.2										
18 *Cyrtodactylusphamiensis*	17.0	16.0	16.4	16.0	14.8	16.4	14.1	14.9	15.1	15.6	16.0	14.4	16.2	17.0	16.0	15.5	16.9									
19 *Cyrtodactylusphukhaensis*	11.4	12.6	17.5	14.6	12.1	14.4	14.9	11.9	14.1	14.0	14.5	13.8	14.1	14.4	7.0	11.2	15.4	15.1								
20 *Cyrtodactyluspuhuensis*	15.4	14.3	18.9	5.7	8.0	7.1	14.4	12.8	14.3	13.5	2.8	13.9	14.9	14.2	14.1	10.5	6.2	15.4	13.7							
21 *Cyrtodactylussolaensis*	16.9	16.5	19.4	17.5	16.9	18.2	16.4	16.7	17.3	14.8	18.0	15.0	16.2	15.9	17.3	16.2	17.7	18.3	16.2	18.0						
22 *Cyrtodactylussoni*	15.5	14.0	18.2	14.6	13.3	14.7	13.9	13.4	14.1	13.4	15.4	7.3	14.1	12.7	14.0	14.3	14.7	14.5	14.0	14.4	15.3					
23 *Cyrtodactylusspelaeus*	16.0	15.5	18.3	10.1	9.3	10.5	15.1	13.5	15.2	13.8	10.5	14.3	15.0	14.5	14.6	11.1	11.3	16.1	13.9	9.1	17.7	14.6				
24 *Cyrtodactylustaybacensis*	17.9	17.4	9.3	16.6	15.6	16.8	15.4	14.8	16.4	16.0	17.0	15.8	17.2	15.6	16.6	16.4	18.3	16.8	16.8	16.3	18.9	15.6	16.1			
25 *Cyrtodactylusvilaphongi*	16.0	13.8	17.8	8.1	7.3	8.2	14.1	13.2	14.2	13.4	8.2	14.2	14.5	14.4	14.0	9.4	9.1	15.0	13.8	7.0	16.9	13.7	9.6	16.3		
26 *Cyrtodactyluswayakonei*	12.1	13.5	18.0	15.5	13.1	15.5	16.2	12.5	15.6	15.3	14.7	15.1	14.2	15.9	7.2	12.2	15.4	15.5	5.0	14.2	16.5	14.0	15.2	17.5	13.7	
27 *Cyrtodactyluszhenkangensis*	9.2	12.8	18.3	14.3	13.2	13.8	14.9	11.8	14.3	12.9	14.0	13.2	15.1	12.9	10.7	13.3	15.5	15.7	10.2	13.3	17.3	13.9	14.1	15.3	13.7	11.9

### ﻿Taxonomy

#### ﻿ Cyrtodactylus nangunhe sp. nov.

Taxon classificationAnimaliaSquamataGekkonidae

A3D638A3-9A23-5C9D-8778-9A5948B5C96D

https://zoobank.org/28F6B348-E2B0-4789-851B-55A9079DD67A

**Figure 2.**
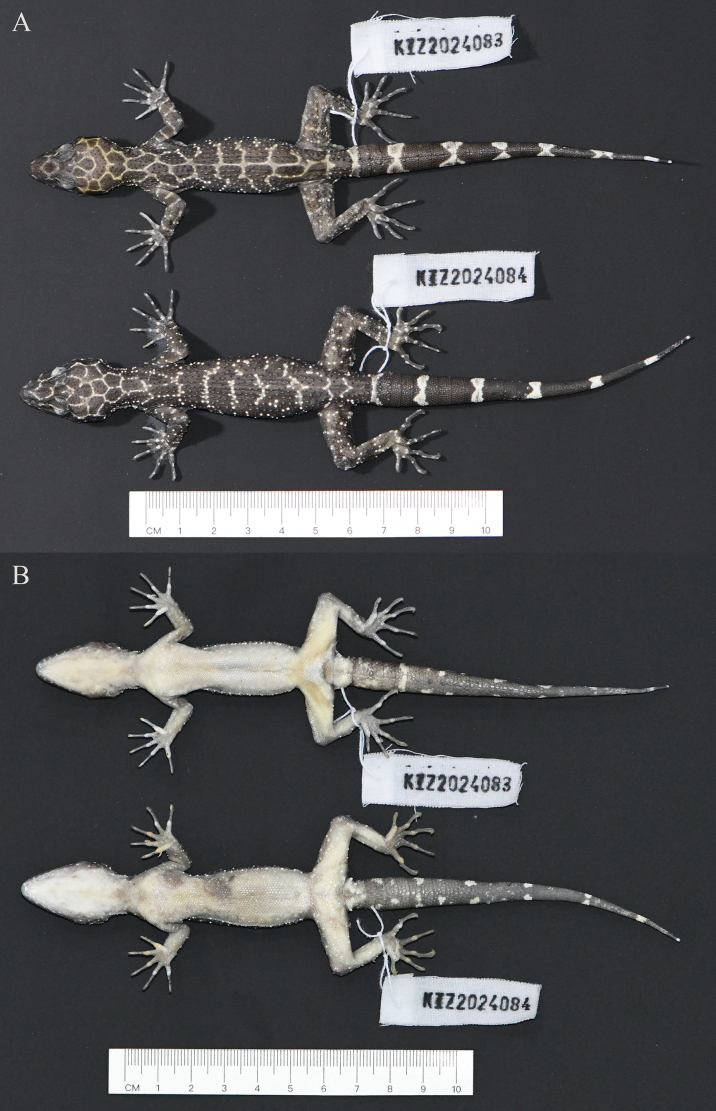
Type specimens of *Cyrtodactylusnangunhe* sp. nov. in preservative **A** dorsal view **B** ventral view.

**Figure 3.**
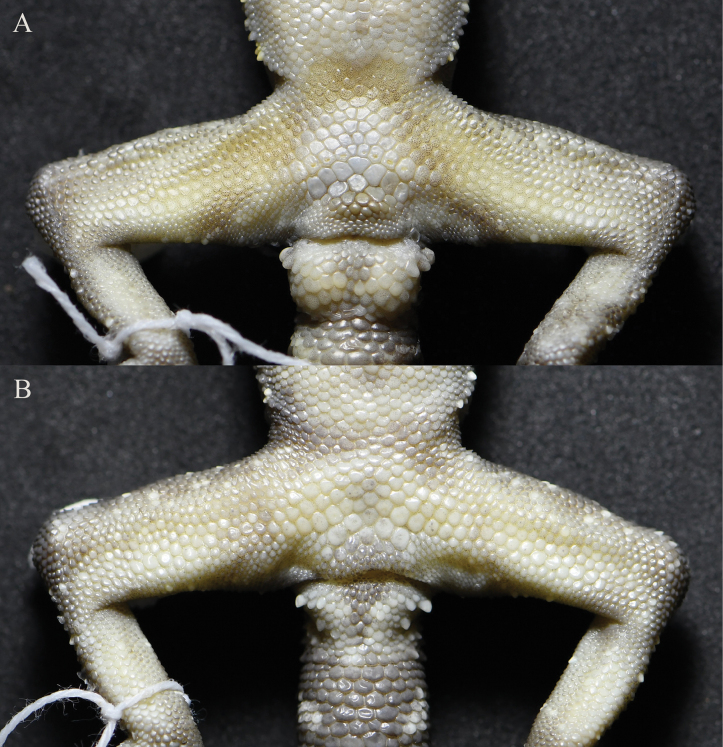
Close-up views of the femoral and precloacal regions of *Cyrtodactylusnangunhe* sp. nov. in preservative **A** male holotype (KIZ 2024083) **B** female paratype (KIZ 2024084).

**Figure 4.**
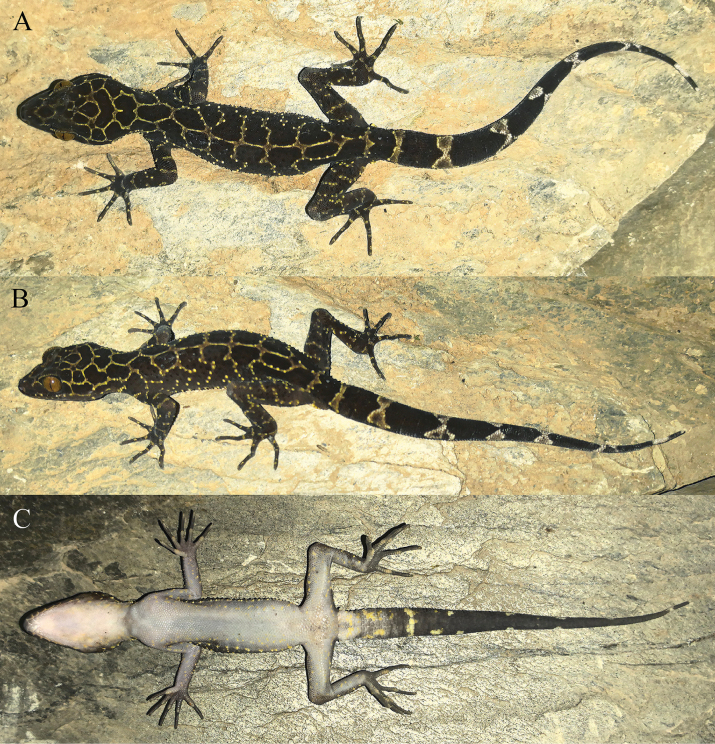
Holotype (KIZ 2024083) of *Cyrtodactylusnangunhe* sp. nov. in life **A** dorsal view **B** lateral view **C** ventral view.

**Figure 5.**
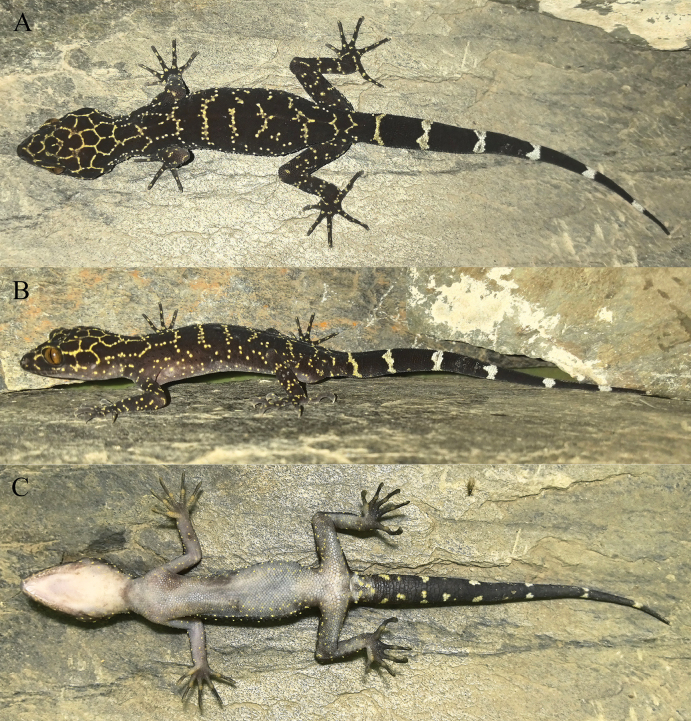
Paratype (KIZ 2024084) of *Cyrtodactylusnangunhe* sp. nov. in life **A** dorsal view **B** lateral view **C** ventral view.

Figs 2 , 3 , 4 , 5 , 7

##### Type material.

***Holotype*.** China • ♂; Yunnan, Cangyuan; 23°13'19"N, 99°1'2"E; 950 m; 17 Aug. 2024; Shuo Liu leg.; KIZ 2024083. ***Paratype*.** China • ♀; same locality; 24 Aug. 2024; Shuo Liu leg.; KIZ 2024084.

##### Diagnosis.

Body size relatively large (SVL 89.5–97.0 mm); tail long (TaL/SVL 1.07–1.14); head relatively long (HL/SVL 0.27–0.28), moderately widened (HW/HL 0.67–0.68); snout long (SE/HL 0.40); body slender (AG/SVL 0.43–0.44); 16–18 longitudinal rows of dorsal tubercles at midbody, 25–27 paravertebral tubercles; ventrolateral fold distinct, interspersed with tubercles; 29–31 longitudinal ventral scale rows at midbody; eight precloacal pores separated by one poreless scale in male; precloacal pores absent, three indistinct shallow pits on enlarged precloacal scales in female; 7–8 slightly enlarged femoral scales beneath each thigh in male, four slightly enlarged femoral scales beneath each thigh in female; enlarged femoral scales separated from enlarged precloacal scales by some smaller scales; femoral pores absent in both sexes; 3–4 shallow pits on enlarged femoral scales on each side in male, absent in female; 1–2 postcloacal tubercles on each side; 19–22 lamellae under finger IV, 24–25 lamellae under toe IV; two rows of subcaudals enlarged; dorsal ground color brownish-black; distinct reticulated pattern composed of thin, light-yellow stripes on dorsal head; six irregular, narrow, light-yellow, transverse bands on dorsum; 6–7 light bands on dorsal tail.

##### Description of holotype.

Adult male, SVL 89.5 mm; head clearly distinguished from neck, relatively long (HL/SVL 0.28), moderately widened (HW/HL 0.67), depressed (HH/HL 0.44); nare oval, surrounded by supranasal dorsally, rostral anteriorly, first supralabial ventrally, and two postnasals posteriorly; snout long (SE/HL 0.40), round anteriorly, longer than diameter of orbit (SE/OD 1.29); snout scales much larger than those in frontal and parietal regions; eye large (OD/HL 0.31), pupils vertical; upper eyelid fringe with spinous scales; ear opening oval, much small in size (ED/HL 0.05); rostral large (RW/HL 0.17), wider than high (RW/RH 1.54), medially divided dorsally by a suture, reaching to approximately half down rostral, in contact with first supralabial laterally on each side and two supranasals and one internasal dorsally; mental triangular, wider than high (MW/ML 1.31), slightly narrower than rostral (MW/RW 0.88); two postmentals, enlarged, in contact posteriorly, bordered by mental anteromedially, first infralabial anterolaterally and one enlarged chin scale posterolaterally on each side, and small chin scales posteriorly; 8/8 supralabials; 8/8 infralabials.

Body slender (AG/SVL 0.42), ventrolateral fold distinct, interspersed with tubercles; dorsal scales granular; dorsal tubercles heterogeneous, conical, in approximately 18 longitudinal rows at midbody, largest ones approximately five times size of adjoining scales and surrounded by 10 granular scales, approximately 25 paravertebral tubercles; gular region with homogenous small smooth scales; ventral scales smooth, homogenous, larger than those of dorsum and in gular region, subimbricate, in approximately 29 longitudinal rows at midbody; precloacal groove absent; precloacal scales significantly enlarged; eight precloacal pores separated by one poreless scale in middle, round or oval; 8/7 slightly enlarged femoral scales, separated from enlarged precloacal scales by some smaller scales; femoral pore absent, four indistinct shallow pits on enlarged femoral scales on left side, three distinct shallow pits on enlarged femoral scales on right side.

Limbs relatively long (ForeaL/SVL 0.16, SL/SVL 0.19), fore limbs slender, hind limbs robust; tubercles on dorsal surface of limbs heterogeneous, conical, ones on fore limbs smaller than most dorsal tubercles, ones on hind limbs approximately as large as most dorsal tubercles; interdigital webbing absent; lamellae under finger IV 21/22, under toe IV 24/24; relative length of fingers I<II≈V<III≈IV, relative length of toes I<II<III<V<IV.

Tail original, long (TaL/SVL 1.14); 2/2 postcloacal tubercles; dorsal tail base with tubercles; subcaudals smooth, two irregular rows enlarged.

##### Color of holotype in life.

Dorsal ground color brownish-black; dorsal surface of head with distinct reticulated pattern composed of thin, light-yellow stripes; nape with irregular thin, light-yellow stripes; dorsum with six irregular, narrow, light-yellow, transverse bands and one longitudinal, continuous, thin, vertebral stripe; flanks scattered with some small light-yellow spots; dorsal surfaces of limbs with indistinct light-yellow bands; dorsal surface of tail black with seven light bands, first two yellowish-gray, last five grayish-white; ventral surface of head pinkish-white, ventral surfaces of body and limbs grayish-white, some light-yellow spots on ventral surfaces of limbs and on ventrolateral surfaces of head and body; ventral surface of tail base gray with some light-yellow spots, other region of ventral tail black; iris bronze.

##### Variations.

Morphometric and meristic data for the type specimens are presented in Table 3. The female paratype (KIZ 2024084) resembles the holotype except that it has no precloacal pores but three indistinct shallow pits on the enlarged precloacal scales, fewer enlarged femoral scales, and no pits on the enlarged femoral scales. Color pattern of the female paratype (KIZ 2024084) also resembles the holotype except that it has no longitudinal vertebral stripe on dorsum and six light bands on the dorsal surface of the tail.

**Table 3.** Measurements (in mm) and meristic data for the type specimens of the new species. Abbreviations defined in Materials and methods.

	KIZ 2024083	KIZ 2024084		KIZ 2024083	KIZ 2024084
	Holotype	Paratype		Holotype	Paratype
	Male	Female		Male	Female
SVL	89.5	97.0	MW	3.8	3.4
TaL	102.2	103.8	ML	2.9	2.9
HH	11.1	11.3	SPL	8/8	9/8
HL	25.2	26.6	IFL	8/8	9/8
HW	17.0	18.2	I	1	1
OD	7.8	8.2	PM	2	2
SE	10.1	10.6	GSDT	10	9
EE	5.2	5.7	DTR	18	16
IND	3.3	3.5	PVT	25	27
IOD	3.2	3.6	V	29	31
ED	1.3	1.5	EFS	8/7	4/4
AG	37.5	42.6	PP	8	0 (3 pitted)
ForeaL	14.3	14.6	FP	0 (4/3 pitted)	0
SL	17.4	17.9	PAT	2/2	1/1
RW	4.3	4.3	LF4	21/22	19/20
RH	2.8	2.6	LT4	24/24	25/24

##### Distribution.

**Figure 6.**
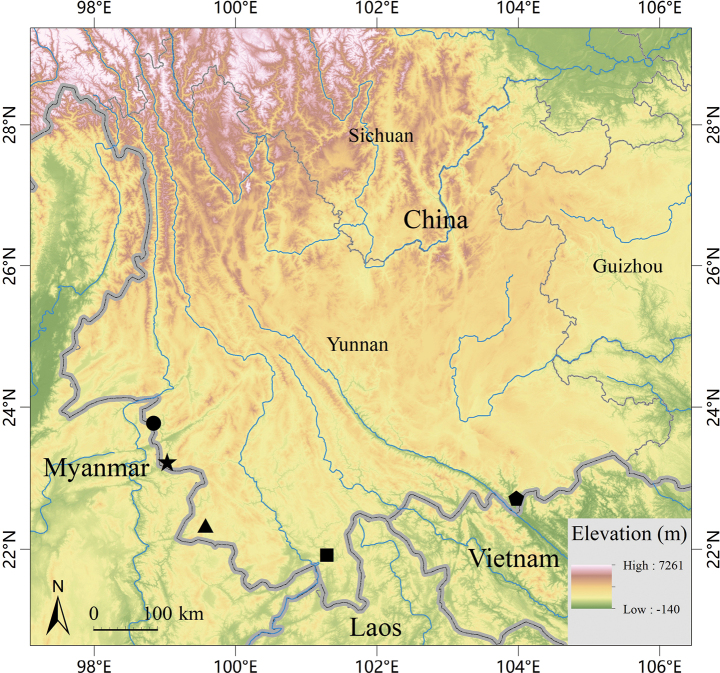
Map showing the distribution of species of the *Cyrtodactyluschauquangensis* group in China. Star, *Cyrtodactylusnangunhe* sp. nov.; dot, *C.zhenkangensis*; triangle, *C.menglianensis*; square, *Cyrtodactyluscaixitaoi*; pentagon, *C.gulinqingensis*.

This species is currently known only from Yunnan Nangunhe National Nature Reserve in Cangyuan County, Yunnan Province, China (Fig. 6).

##### Natural history.

**Figure 7.**
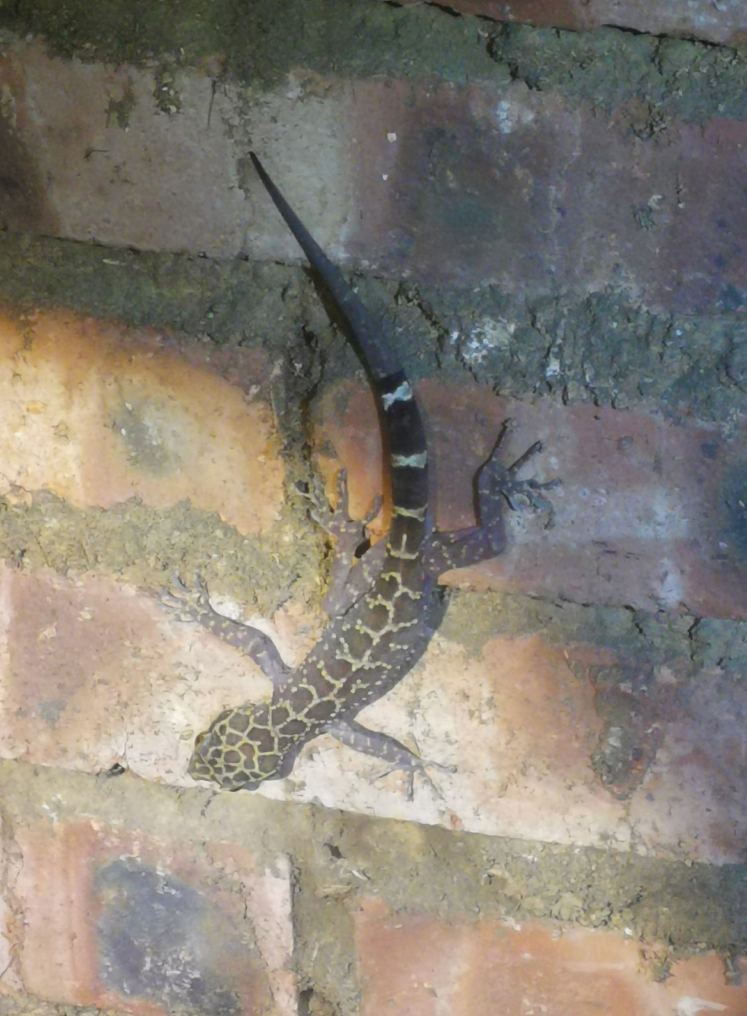
*Cyrtodactylusnangunhe* sp. nov. from near the type locality.

There is no karst landform in the area where the type specimens were collected. This species was found on rocks or tree trunks in the virgin evergreen broadleaved forest at night. Individuals were slow and easy to catch. In addition, this species was found on the wall of an abandoned house near the collection site of the type specimens by locals (Fig. 7). The only female specimen did not carry eggs and no juveniles were found, so the reproductive season of this species is unknown. Other reptiles found at the type locality of the new species include *Acanthosaurarubrilabris* Liu, Rao, Hou, Orlov, Ananjeva & Zhang, 2022, *Boigamultomaculata* (Boie, 1827), *Calotesemma* Gray, 1845, *Hemidactylusgarnotii* Duméril & Bibron, 1836, *Lycodonfasciatus* (Anderson, 1879), and *Ptyaskorros* (Schlegel, 1837).

##### Etymology.

The specific epithet *nangunhe* is a noun in apposition, and therefore invariable; it refers to Yunnan Nangunhe National Nature Reserve, where the new species was found.

##### Comparisons.

*Cyrtodactylusnangunhe* sp. nov. can be distinguished from all other members of the *C.chauquangensis* species group by a unique combination of morphological characters. *Cyrtodactylusnangunhe* sp. nov. differs from *C.auribalteatus* Sumontha, Panitvong & Deein, 2010, *C.bichnganae* Ngo & Grismer, 2010, *C.doisuthep* Kunya, Panmongkol, Pauwels, Sumontha, Meewasana, Bunkhwamdi & Dangsri, 2014, *C.dumnuii* Bauer, Kunya, Sumontha, Niyomwan, Pauwels, Chanhome & Kunya, 2010, *C.erythrops* Bauer, Kunya, Sumontha, Niyomwan, Panitvong, Pauwels, Chanhome & Kunya, 2009, *C.gulinqingensis*, *C.huongsonensis* Luu, Nguyen, Do & Ziegler, 2011, *C.kunyai* Pauwels, Sumontha, Keeratikiat & Phanamphon, 2014, *C.luci* Tran, Do, Pham, Phan, Ngo, Le, Ziegler & Nguyen, 2024, *C.ngoiensis* Schneider, Luu, Sitthivong, Teynié, Le, Nguyen & Ziegler, 2020, *C.phamiensis* Grismer, Aowphol, Grismer, Aksornneam, Quah, Murdoch, Gregory, Nguyen, Kaatz, Bringsøe & Rujirawan, 2024, *C.phukhaensis* Chomdej, Pradit, Pawangkhanant, Naiduangchan & Suwannapoom, 2022, *C.soni* Le, Nguyen, Le & Ziegler, 2016, and *C.sonlaensis* Nguyen, Pham, Ziegler, Ngo & Le, 2017 by the absence of femoral pores (vs femoral pores present).

*Cyrtodactylusnangunhe* sp. nov. differs from *C.bobrovi* Nguyen, Le, Pham, Ngo, Hoang, Pham & Ziegler, 2015, *C.chauquangensis* Hoang, Orlov, Ananjeva, Johns, Hoang & Dau, 2007, *C.houaphanensis* Schneider, Luu, Sitthivong, Teynié, Le, Nguyen & Ziegler, 2020, *C.menglianensis*, *C.otai* Nguyen, Le, Van Pham, Ngo, Hoang, The Pham & Ziegler, 2015, *C.spelaeus* Nazarov, Poyarkov, Orlov, Nguyen, Milto, Martynov, Konstantinov & Chulisov, 2014, and *C.wayakonei* by having enlarged femoral scales (vs femoral scales not enlarged).

*Cyrtodactylusnangunhe* sp. nov. differs from *C.caixitaoi* by having different dorsal coloration (brownish-black ground color with thin stripes on head and narrow bands on dorsum vs orange brown or pinkish-brown ground color with thick stripes on head and wide bands on dorsum), more longitudinal rows of dorsal tubercles at midbody (16–18 vs 14–15), and more paravertebral tubercles (25–27 vs 20–21).

*Cyrtodactylusnangunhe* sp. nov. differs from *C.cucphuongensis* Ngo & Chan, 2011 by the different conditions of precloacal pores in males (present vs absent).

*Cyrtodactylusnangunhe* sp. nov. differs from *C.martini* Ngo, 2011 by having more precloacal pores in males (eight vs four), fewer longitudinal ventral scale rows (29–31 vs 39–43), and enlarged femoral scales separated from enlarged precloacal scales by smaller scales (vs enlarged femoral scales continuous with enlarged precloacal scales).

*Cyrtodactylusnangunhe* sp. nov. differs from *C.puhuensis* Nguyen, Yang, Le, Nguyen, Orlov, Hoang, Nguyen, Jin, Rao, Hoang, Che, Murphy & Zhang, 2014 by having more precloacal pores in males (eight vs five), enlarged femoral scales separated from enlarged precloacal scales by smaller scales (vs enlarged femoral scales continuous with enlarged precloacal scales), and two rows of subcaudals enlarged under original tail (vs one row).

*Cyrtodactylusnangunhe* sp. nov. differs from *C.taybacensis* Pham, Le, Ngo, Ziegler, Nguyen, 2019 by having fewer precloacal pores in males (eight vs 11–13), ventrolateral fold with interspersed tubercles (vs without), and two rows of subcaudals enlarged under original tail (vs one row).

*Cyrtodactylusnangunhe* sp. nov. differs from *C.vilaphongi* Schneider, Nguyen, Duc Le, Nophaseud, Bonkowski & Ziegler, 2014 by being larger (SVL 89.5–97.0 mm vs 60.9–86.1 mm), dorsal head with a distinct reticulated pattern (vs indistinct), and having enlarged subcaudals (vs not enlarged).

*Cyrtodactylusnangunhe* sp. nov. differs from its sister species *C.zhenkangensis* by enlarged femoral scales separated from enlarged precloacal scales by smaller scales (vs enlarged femoral scales continuous with enlarged precloacal scales), femoral pores absent (vs femoral pores present), having fewer pitted precloacal scales in females (three vs 7–9), fewer longitudinal rows of dorsal tubercles at midbody (16–18 vs 20–24), more lamellae under toe IV (24–25 vs 21–23), thin stripes on head and narrow bands on dorsum (vs thick stripes on head and wide bands on dorsum), fewer light bands on tail (6–7 vs 8–10), and most light bands on tail not connected on ventral surface of tail (vs connected on ventral surface of tail).

## ﻿Discussion

Grismer et al. (2021) partitioned the species of *Cyrtodactylus* into 10 ecotypes according to their habitat preferences. The *C.chauquangensis* group is a karst ecotype group, and most species of this group are karst dwellers except for *C.doisuthep* and *C.phukhaensis*, which inhabit the forests in northern Thailand (Kunya et al. 2014; Chomdej et al. 2022; Tran et al. 2024). *Cyrtodactylusnangunhe* sp. nov. represents the third forest-dwelling species of this group. Although Cangyuan County is rich in karst landforms, we have not found any individual of *Cyrtodactylus* in the karst habitats in this county. On the contrary, there is no karst landform in the area where *C.nangunhe* sp. nov. was discovered. The reason is unknown why this species does not live in karst areas but instead in the forest without karst habitat.

The straight-line distance between the type localities of *C.nangunhe* sp. nov. and its sister species *C.zhenkangensis* is only approximately 65 km. However, these two species inhabit very different habitats: *C.zhenkangensis* in an area of limestone and *C.nangunhe* sp. nov. in forest. Limestone is usually grayish-white and has many cracks and holes. Individuals of *C.zhenkangensis* have a light general color and relatively distinct stripes on the dorsal surface, which may be to better hide themselves in limestone environments. On the other hand, the rocks or tree trunks in forests are often of a single color, and living in forests often requires passing on the ground. Individuals of *C.nangunhe* sp. nov. have a dark general color and relatively indistinct stripes on the dorsal surface, which may make them less visible to predators in the forest. Perhaps it is the differentiation of habitats that has led to the genetic divergence between *C.zhenkangensis* and *C.nangunhe* sp. nov. Although most species of this genus tend to inhabit karst habitats, some of them can adapt to other habitats such as forests. The discovery of the new species reminds us that the diversity of forest-dwelling species of this genus is still greatly underestimated.

## Supplementary Material

XML Treatment for Cyrtodactylus nangunhe

## ﻿Additional information

### Author ORCIDs

Shuo Liu https://orcid.org/0000-0001-7825-3006

Mian Hou https://orcid.org/0000-0002-3322-6570

Dingqi Rao https://orcid.org/0000-0003-2680-1503

### Data availability

All of the data that support the findings of this study are available in the main text.
